# Efficacy of BCG vaccination against COVID-19 in health care workers and non-health care workers: A meta-analysis of randomized controlled trials

**DOI:** 10.1371/journal.pone.0321511

**Published:** 2025-05-13

**Authors:** Zhuoyang Xia, Jiahao Meng, Xuanyu Wang, Pan Liu, Yumei Wu, Yilin Xiong, Baimei He, Shuguang Gao

**Affiliations:** 1 Department of Orthopaedics, Xiangya Hospital, Central South University, Changsha, Hunan, China; 2 Xiangya School of Medicine, Central South University, Changsha, Hunan, China; 3 Key Laboratory of Aging-related Bone and Joint Diseases Prevention and Treatment, Ministry of Education, Xiangya Hospital, Central South University, Changsha, China; 4 National Clinical Research Center of Geriatric Disorders, Xiangya Hospital, Central South University, Changsha, Hunan, China; 5 Department of Geriatric Respiratory and Critical Care Medicine, Xiangya Hospital, Central South University, Changsha, China; Universitas Padjadjaran, INDONESIA

## Abstract

**Background:**

The Bacillus Calmette-Guérin (BCG) vaccine has shown potential non-specific protection against infectious diseases through “trained immunity”, which may offer cross-protection against viral infections. However, there is no consensus on whether BCG vaccination could prevent COVID-19 or reduce its symptoms.

**Methods:**

PubMed, Cochrane Library, Embase and Web of Science were searched for randomized controlled trials on BCG vaccination and COVID-19 prevention, covering studies from the inception of each database to 2 May 2024. We included studies where participants, not infected with COVID-19, were vaccinated with BCG or placebo. We excluded non-randomized trials, studies without full texts, unrelated interventions, and those not reporting relevant outcomes. Clinical data on COVID-19 infection, severity, hospitalization, mortality, and other adverse events, were extracted and analyzed. The DerSimonian–Laird random-effects model and the Cochrane Collaboration’s risk of Bias Tool were used for analysis and risk of bias assessment.

**Results:**

A total of 12 RCTs involving 18,086 patients were finally included. For the prophylactic effect of BCG on COVID-19, pooled results showed no statistically significant difference between BCG and placebo (pooled RR 1.02; 95%CI: 0.91–1.14). There was no statistically significant difference between non-health care workers (pooled RR 0.91; 95%CI: 0.67–1.24) and health care workers (pooled RR 1.03; 95%CI: 0.93–1.15). Regarding COVID-19 severity, no significant difference were found for asymptomatic (pooled RR 1.18; 95%CI: 0.81–1.72), mild to moderate (pooled RR 0.99; 95%CI: 0.84–1.17), severe COVID-19 (pooled RR 1.25; 95%CI: 0.92–1.70), hospitalization (pooled RR 0.93; 95%CI: 0.58–1.50) or all-cause mortality (pooled RR 0.60; 95%CI: 0.18–1.95) between BCG and placebo groups. Subgroup analysis also showed no significant difference between BCG and placebo in non-health care workers or health care workers.

**Conclusions:**

Vaccination of BCG could not effectively prevent COVID-19 infection or decrease COVID-19 symptoms both in non-health care workers and health care workers.

## Introduction

During the COVID-19 pandemic, a number of people tragically deceased due to COVID-19 infection. As of the 31st of December 2023, there have been more than 773 million cases of COVID-19 reported to the World Health Organization (WHO), with over 7 million deaths due to COVID-19 [1]. The spatial and temporal heterogeneity in the emergence, transmission, and nature of new coronaviruses poses a great challenge for COVID-19 vaccination. Previously, some meta-analyses for COVID-19 prevention [2–4] were conducted.

The Bacillus Calmette-Guérin (BCG) vaccine is primarily used to prevent tuberculosis, but in recent years, it has gained widespread attention for its potential non-specific protective effects against a range of infectious diseases. This phenomenon is closely related to the concept of “trained immunity”, which refers to long-lasting changes in the innate immune system induced by vaccination or microbial exposure, thereby enhancing the body’s ability to respond to a broad array of infections. BCG vaccination may provide cross-protection against viral infections, particularly respiratory viral infections, by enhancing the responsiveness of immune cells such as monocytes and macrophages [5]. With the outbreak of the COVID-19 pandemic, BCG has attracted significant interest as a potential adjunctive strategy to reduce COVID-19 infection and its severity.

Epidemiologic studies have suggested a negative association between national bacillus Calmette-Guérin (BCG) vaccination policy and the prevalence and mortality of COVID-19 [6], revealing that BCG vaccine may act as a protective factor against infection and death associated with COVID-19.

There is no clear consensus on whether BCG vaccination prevents COVID-19 infection and reduces mortality. A randomized controlled trial (RCT) showed that BCG vaccination was effective in protecting against COVID-19 infection compared with placebo [7]. While the study group was changed to health care workers, a recent RCT (n = 1221) showed that BCG vaccine did not enhance the protection against COVID-19 infection [8].

We hypothesized that there may be differences in the protective efficacy of BCG against COVID-19 in the non-health care workers versus health care workers. Our hypothesis is based on three reasons. Firstly, health care workers are exposed to higher viral loads and higher frequencies of pathogens due to the nature of their occupation, potentially leading to a stronger response from the immune system. BCG vaccination may help to reduce the risk of post-exposure infections or attenuate conditions by inducing a stronger immune response. Secondly, health care workers typically have greater immune alertness and awareness of the disease and may be able to stimulate their immune system more effectively following vaccination. Thirdly, health care workers may already have a strong immune background due to frequent occupational exposures (e.g., prior vaccinations, infections, etc.), and these factors may enhance the efficacy of BCG.

Therefore, we suggest that BCG may perform better in health care workers, mainly because their immune system is already in a state of “high alert” through prolonged occupational exposures and is able to respond more efficiently to the “trained immunity” triggered by the BCG vaccine. BCG may provide enhanced immune protection for health care workers, especially against viral diseases such as COVID-19.

Previous meta-analysis did not analyze both the non-health care workers group and the health care workers group, which concluded that BCG vaccination was not effective in protecting the population against COVID-19 infection [9]. In addition, some of the latest RCTs [8,10–12] were not included, so the assessment of BCG vaccination required further updating. Therefore, we conducted an updated meta-analysis to further evaluate the efficacy of BCG against COVID-19 by comparing its protective effects on the non-health care workers and health care workers respectively, which could help guide the practice of prevention and treatment of COVID-19.

## Methods

This meta-analysis was performed according to the Cochrane guidelines and the Preferred Reporting Items for Systematic Reviews and Meta-Analyses (PRISMA) guidelines, which has been reported in line with the Assessing the methodological quality of systematic reviews (AMSTAR) Guidelines [13,14]. The International Prospective Register of Systematic Reviews (PROSPERO) protocol is available online (https://www.crd.york.ac.uk/prospero/display_record.php?ID=CRD42024566212).

### Search strategy and selection criteria

Comprehensive literature search for studies on whether BCG vaccination could prevent against COVID-19 was conducted in the electronic databases PubMed, Cochrane Library, Embase, and Web of Science. We searched studies published until 2 May 2024. After removing duplicate articles, three reviewers (ZYX, JHM, and XYW) independently screened the titles and abstracts to identify eligible RCTs. We included studies in which people who were not infected with COVID-19 were respectively vaccinated with BCG and placebo (or no vaccination), and whose primary outcome metric was the rate of COVID-19 infection, secondary outcome metrics included COVID-19 severity (defined by WHO, https://apps.who.int/iris/handle/10665/338882), hospitalization associated with COVID-19, and all-cause mortality. We did not limit the languages used for the study, which included English and other languages. We excluded non-randomized controlled trials, studies for which the full text could not be obtained, studies with not related intervention, and studies that did not report relevant outcomes. Endnote X9 was used to screen title abstracts and the studies’ full text. Any discrepancies were resolved after discussion with a senior author.

### Data extraction

The four reviewers (ZYX, JHM, XYW and PL) independently extracted pertinent data from the included studies using a standardized form. Excel was used for extraction. This form included the following details: first author, year of publication, country, number of intervention and control groups, average (median) age, percentage of female, intervention and control, study population, diagnosis of COVID-19, follow-up duration, and the aimed outcomes. In cases of missing data, efforts were made to contact the corresponding authors for the original information. Any disagreements were resolved through discussion.

### Quality assessment

The same four reviewers independently assessed the included studies for the risk of bias using the Cochrane Collaboration’s risk of Bias Tool. This tool consists of seven domains of bias (random sequence generation, allocation concealment, blinding of participants and personnel, blinding of outcome assessment, incomplete outcome data, selective reporting, and other bias), and each domain was rated as of a low, unclear, or high risk of bias. Ultimately, the overall bias of each study was classified as either low risk, some concerns, or high risk, based on the comprehensive assessment of bias across these domains. Discrepancies, if any, were resolved by discussion.

### Statistical analysis

Binary variables were presented as event number and total number. The results were presented as pooled risk ratio (RR) with 95% confidence interval (CI). Heterogeneity refers to the variability that exists between multiple independent studies or data sets. Considering the potential clinical heterogeneity arising from the types of BCG vaccines and the injection methods, we used the DerSimonian–Laird random-effects model for analysis. Heterogeneity analysis was tested by Q-statistic (P < 0.1), and I^2^-statistic (I^2^ > 50%). Based on the different exposure risks, we categorized participants into non-health care workers and health care workers for subgroup analysis. The pooled RRs were presented. We conducted sensitivity analyses using leave-one-out method. To explore the impact of study population (country), follow-up duration, and different types of BCG strains on heterogeneity, a meta-regression analysis was performed on these characteristics. Funnel plot and Egger’s test were used to assess the publication bias. We evaluated the strength of evidence using the GRADE (Grading of Recommendations Assessment, Development and Evaluation) system [15,16]. All statistical analyses were performed using Review Manager 5.4 and Stata17.

## Results

### Literature search

**Fig 1:**
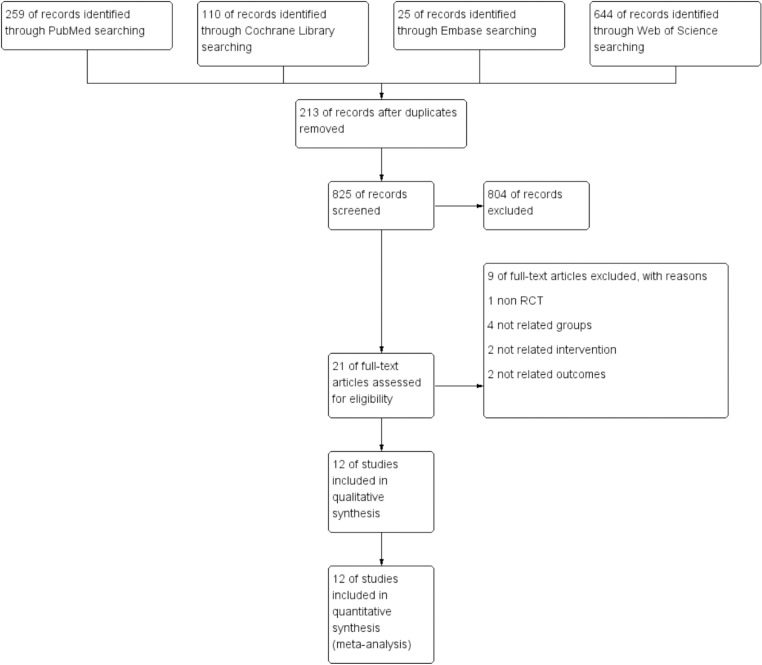
Flowchart of literature retrieval.

The flow diagram of the literature search and study selection are shown in Fig 1. Through the retrieval formula, 825 potentially relevant citations were retrieved. After screening the titles and abstracts with the retrieved full text, 21 studies were included. These studies were then evaluated in full text and 9 studies were removed based on the inclusion criteria. In the end, 12 RCTs [7,8,10–12,17–23] (including 5 RCTs on non-health care workers and 7 RCTs on health care workers) with a total of 18086 participants were included in the final analysis.

### Baseline study characteristics

Table 1 shows the baseline characteristics of the included RCTs. All the included RCTs were balanced in terms of demographic data between the intervention and control groups. The study population was mainly categorized into non-health care workers groups (n = 5) and health care workers groups (n = 7). One in non-health care workers groups consisted of adults with fundamental diseases, including diabetes or diabetes-related complications, chronic kidney disease, chronic lung disease, etc, who were thereby at higher risk of severe COVID-19 infection [20]. The intervention group was injected with 0.1 ml of BCG vaccine of Danish strain, Moreau strain, Moscow, or other types. The control group was injected with 0.1 ml of saline as placebo or not treated. Follow-up period ranged from three to twelve months.

**Table 1:** Study characteristics.

Study	Year	Country	Intervention number	Control number	Average (median) age		Female (%)		Intervention	Control	Population	COVID-19 diagnosis	Follow-Up duration
					Intervention	Control	Intervention	Control					
Tsilika	2022	Greece	148	153	68.6	68.7	33.8	30.3	0.1 mL BCG Moscow	Placebo	Non-health care workers (elderly)	Positive PCR	6 months
Koekenbier	2023	Netherlands	3058	3054	69 (median)	69 (median)	36.8	37.7	0.1 mL BCG Danish strain 1331	Placebo	Non-health care workers (elderly)	Self-report	6 months
Doesschate	2022	Netherlands	753	758	41.3	42.8	76	72.6	0.1 mL BCG Danish strain 1331	Placebo	Health care workers	Positive PCR	12 months
Claus	2023	Netherlands	665	644	41.79	43.21	75.4	73.4	BCG	Placebo	Health care workers	Self-report, serologic test, or symptoms	12 months
Madsen	2024	Denmark	610	611	48 (median)	47 (median)	83.1	82.6	0.1 mL BCG Danish strain 1331	Placebo	Health care workers	Self-report	6 months
Santos	2023	Brazil	134	130	NA	NA	76.9	82.3	0.1 mL BCG Moreau strain	Placebo	Non-health care workers	Positive PCR or serologic test	6 months
Dos Anjos	2022	Brazil	64	67	41.8	44.2	68.8	83.6	0.1 ml BCG Moscow	No treatment	Health care workers	Positive PCR or rapid antigen test	6 months
Sinha	2023	India	246	249	43	44	50.4	45.4	0.1 mL BCG	Placebo	Non-health care workers (adults with fundamental diseases)	Positive PCR	9 months
Moorlag	2022	Netherlands	1008	1006	67 (median)	67 (median)	48.8	46.1	0.1 mL BCG Danish strain 1331	Placebo	Non-health care workers (elderly)	Positive PCR	12 months
Czajka	2022	Polland	168	174	42.2	46.3	74.8	75.6	BCG	Placebo	Health care workers	Positive PCR	3 months
Pittet	2023	Australia, Netherlands, Spain, UK, and Brazil	1703	1683	42.8	42.8	73.1	76.1	0.1 mL BCG Danish strain 1331	Placebo	Health care workers	Positive PCR, rapid antigen test, or serologic test	6 months
Upton	2024	South Africa	500	500	39 (median)	39 (median)	69.8	71	0.1 mL BCG Danish strain 1331	Placebo	Health care workers	Positive PCR	12 months

BCG, Bacillus Calmette-Guérin; PCR, Polymerase Chain Reaction.

### Quality assessment

**Fig 2:**
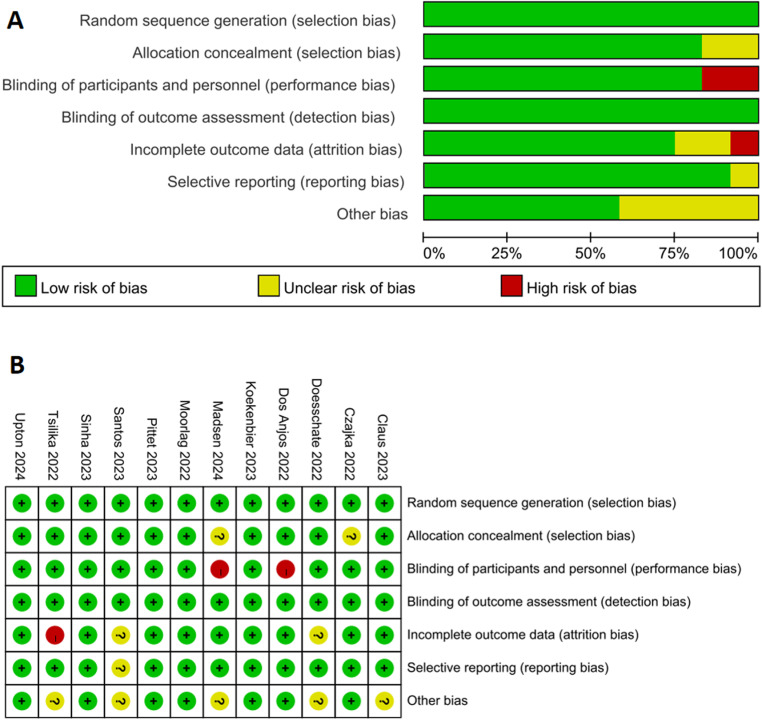
Graph of the risk of bias for the included randomized controlled trials (A); Summary of the risk of bias for the included randomized controlled trials (B).

Six of the twelve RCTs [10,12,18,20,21,23] were rated as of a low risk. One RCT [19] did not use a placebo for health care workers allocated to the unvaccinated group and, furthermore, the health care workers would easily determine that they were in the placebo group. One RCT [8] employed a single-blind design, where only the participants were blinded to the treatment provided, was rated as high risk in terms of blinding of participants and personnel. Another RCT [7] had a high risk of bias for the lack of microbiological evidence for COVID-19 diagnosis. Because of the high likelihood of COVID-19 infection from other alternative diagnoses, this meta-analysis included the number of all individuals suspected of being infected with COVID-19 in the RCT as the rate of COVID-19 infection. Fig 2 shows the distribution of bias.

### Preventive role of BCG on COVID-19

**Fig 3:**
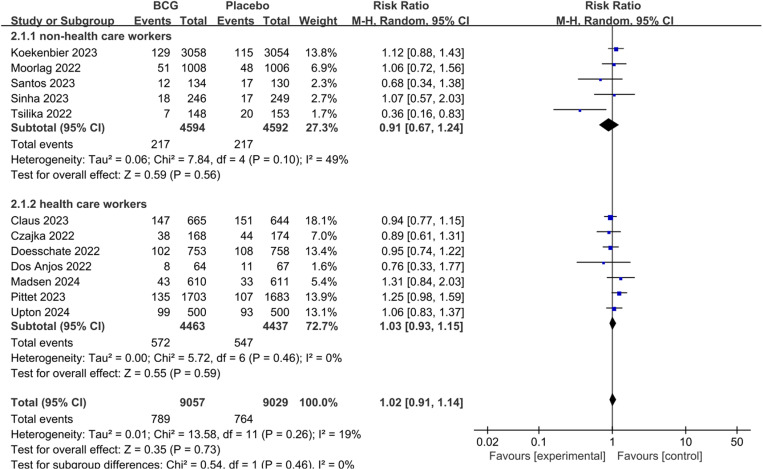
The pooled effect of COVID-19 infection rate in non-health care workers and health care workers.

A total of 12 studies reported the prophylactic effect of BCG on COVID-19, and the pooled results showed there was no statistically significant difference between BCG group and placebo group (pooled RR 1.02; 95%CI: 0.91–1.14) nor was a statistically significant difference found between non-health care workers (pooled RR 0.91; 95%CI: 0.67–1.24) and health care workers (pooled RR 1.03; 95%CI: 0.93–1.15) based on different population analyses (Fig 3).

**Fig 4:**
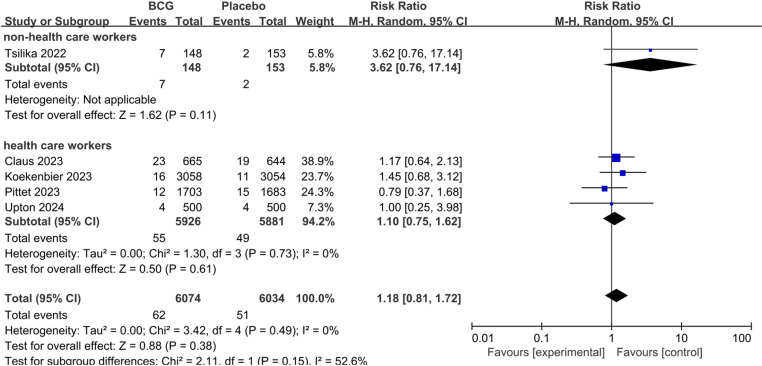
The pooled effect of COVID-19 asymptomatic infection rate in non-health care workers and health care workers.

**Fig 5:**
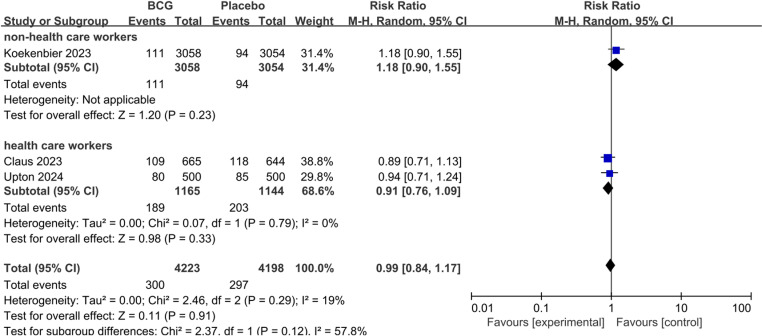
The pooled effect of COVID-19 mild or moderate infection rate in non-health care workers and health care workers.

**Fig 6:**
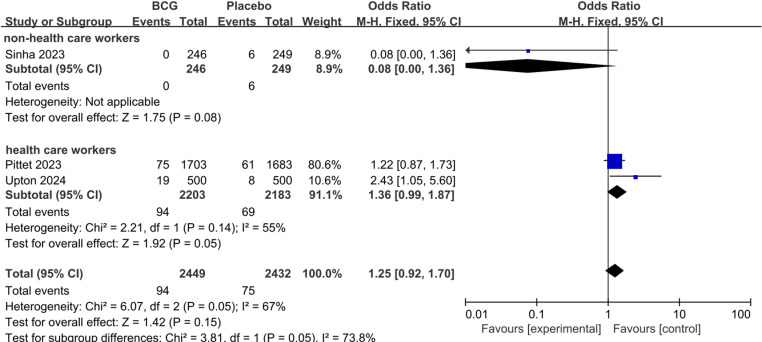
The pooled effect of COVID-19 severe infection rate in non-health care workers and health care workers.

We further assessed the role of BCG for COVID-19 severity. There was no statistically significant difference between BCG and Placebo on the rate of asymptomatic COVID-19 (pooled RR 1.18; 95%CI: 0.81–1.72), mild to moderate COVID-19 (pooled RR 0.99; 95%CI: 0.84–1.17), and severe COVID-19 (pooled RR 1.25; 95%CI: 0.92–1.70). We performed subgroup analyses based on non-health care workers and health care workers, also did not find statistically significant differences between the groups (Figs 4–6).

**Fig 7:**
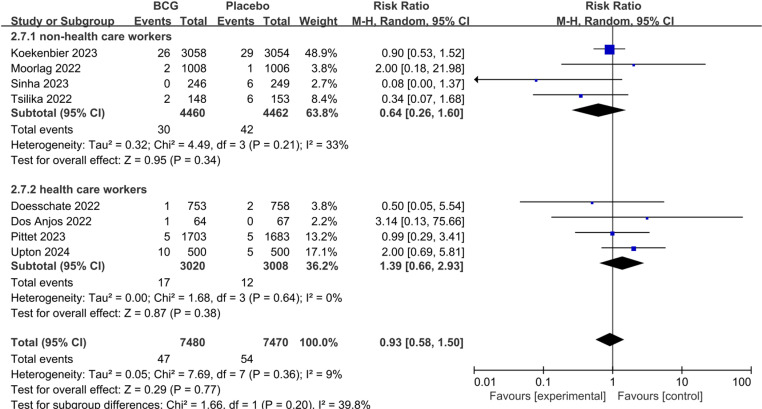
The pooled effect of hospitalization rate associated with COVID-19 in non-health care workers and health care workers.

**Fig 8:**
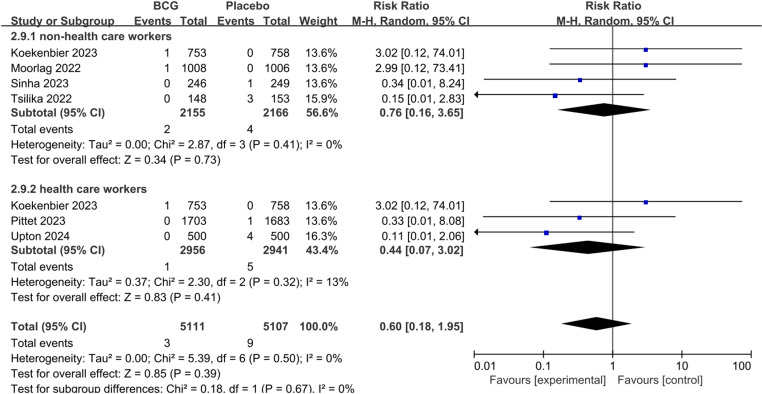
The pooled effect of all-cause mortality rate in non-health care workers and health care workers.

With respect to hospitalization associated with COVID-19 (pooled RR 0.93; 95%CI: 0.58–1.50) and all-cause mortality (pooled RR 0.60; 95%CI: 0.18–1.95), there was no statistically significantly difference in both groups (Figs 7,8).

### Sensitivity analysis

We analyzed the above results in subgroups based mainly on the exposure risk of the patients, i.e., whether they were health care workers or not, and did not find any statistical differences in the individual outcomes. The leave-one-out method was also used to assess the robustness of the above outcomes. The results indicated that excluding any individual study did not significantly affect the stability of the overall outcomes. Meta-regression analyses were performed separately based on study population (country), follow-up duration, and different types of BCG strains, and no significant impact of these factors on heterogeneity was found.

### Publication bias

**Fig 9:**
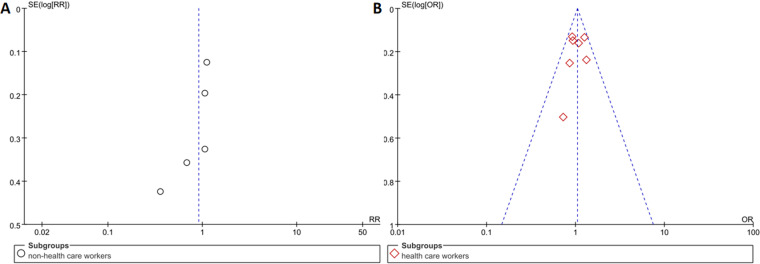
The funnel plot based on COVID-19 infection in non-health care workers (A) and health care workers (B).

For the primary outcome (BCG preventive effect), we plotted the funnel plot (Fig 9) and the results showed symmetry. We also performed the Egger’s test, and no significant publication bias was found (p > 0.05).

### GRADE of evidence

We scored the above outcomes according to the GRADE system, and the quality of evidence for outcomes varied from low quality to high quality. COVID-19 infection and mild to moderate infection of health care workers are of high quality.

## Discussion

This meta-analysis showed that neither non-health care workers nor health care workers vaccinated with BCG has the ability to combat COVID-19 with more efficacy, but this does not mean that BCG had an equal effect on these two groups. Most of the Risk Ratio values for the outcome measures were higher and greater than 1 for health care workers, while most of which were less than 1 for the non-health care workers groups, implying that BCG may indeed have opposite effects on the two groups.

For the assessment of whether vaccination of BCG could prevent COVID-19 infection or decrease COVID-19 symptoms, most of the 12 randomized controlled trials described above looked at the extent of COVID-19 infections, COVID-19 severity (asymptomatic, mild, moderate, severe infection), hospitalization/ICU/death associated with COVID-19, all-cause hospitalization/ICU/death, and other adverse events. These outcome metrics enabled a more accurate assessment of the extent of prevention, control, and prognosis of COVID-19.

Basic research demonstrated the ineffectiveness of BCG vaccine. Kaufmann et al. [24] found that BCG failed to provide protection against SARS-CoV-2, whose infection led to distinctive pulmonary vascular damage that facilitated viral dissemination to other organs, including bone marrow, which was the centerpiece of BCG-mediated training immunity. Monocytes from BCG-vaccinated individuals produced an ineffective cytokine response to SARS-CoV-2.

Our current evidence is insufficient to support BCG vaccination program to prevent COVID-19 infection, consistent with the WHO recommendation against BCG vaccination to prevent COVID-19 in 2020 [25]. Adopting a program of BCG vaccination to prevent COVID-19 infection may not be a good option in settings with limited medical resources. At this time, the COVID-19 vaccines, which mainly include BNT162b2 vaccine (Pfizer-BioNTech), mRNA-1273 (Moderna), ChAdOx1 nCoV-19 (AstraZeneca), and Janssen (Johnson & Johnson), which have received early regulatory authorization based on their efficacy [26]. They are currently recognized as the main vaccines against COVID-19 infection.

This meta-analysis included additional large-sample RCTs after the publication of previous meta-analysis, and the additional RCTs were mainly in 2023–2024. In addition, due to the heterogeneity of the work environment and intensity of the work of non-health care workers s and health care workers this meta-analysis was subdivided into two subgroups to explore whether there were differences between these two different populations. This meta-analysis was more rigorous with respect to the inclusion and exclusion criteria than previous meta-analysis so that the literature with higher quality was included. One study by Faustman et al. [27] with a study population of patients with long-standing type I diabetes and an intervention of vaccination with multiple doses of BCG differed significantly from the other studies and was therefore not included in this meta-analysis. Another study by Blossey et al. [28] involved an intervention of vaccination with VPM1002 (a genetically modified BCG vaccine). This vaccine may have elicited immune responses that differed significantly from those induced by the standard BCG vaccine, and therefore, this study was not included in the meta-analysis. The outcome measures increased the severity of COVID-19 symptoms, which helped to better assess the progression of COVID-19 disease.

There are several limitations in our study. Firstly, in the included literature, three RCTs’ assessments of outcome indicators during follow-up were based on self-report, which may be somewhat biased [8,10,18]. Secondly, the number of included studies was small, so there were not sufficient literature data to support some outcome measures (e.g., COVID-19 severity). Thirdly, the included studies were conducted in different countries (e.g., Brazil, India, Greece, Netherlands, Denmark, etc.) and there was heterogeneity in the study populations. Fourth, the duration of follow-up is heterogeneous, ranging from three to twelve months, and it is possible that the number of most vaccine-derived antibodies declines over time, potentially reducing the effectiveness of the BCG vaccination [29]. Also, these study populations were inoculated with different types of BCG (e.g., Danish strain, Moreau strain, etc.) and the interventions were heterogeneous. It has been suggested that different types of BCG strains may exhibit different virulence and thus different levels of effectiveness in disease prevention and progression [30]. Clinical heterogeneity based on the aforementioned characteristics was observed across studies. However, no significant impact of these factors on heterogeneity was found through meta-regression, which may be attributed to the limited number of studies included. Future research with a larger sample size is needed to provide higher-quality evidence. Lastly, this meta-analysis only assessed the efficiency of BCG vaccines, and there was a large heterogeneity for its safety-related outcome metrics, which were therefore not included in this evaluation.

## Conclusions

Based on the current studies, it cannot be demonstrated that vaccination of BCG could effectively prevent COVID-19 infection and decrease COVID-19 symptoms, hospitalization rate and mortality rate both in non-health care workers and health care workers. However, more high-quality studies are needed to further confirm the efficacy of BCG against COVID-19.

## Supporting information

S1 Checklist PRISMA_2020_checklist. (DOCX)

S2 Checklist PRISMA_2020_abstract_checklist. (DOCX)

S1 File Search strategy. (DOCX)

S2 File Literature search. (XLSX)

S3 File Data extraction. (XLSX)

S4 File Cochrane Collaboration’s risk of bias tool. (XLSX)

S5 File Grade system. (XLSX)
