# Supplementary material for: Efficacy of BCG vaccination against COVID-19 in health care workers and non-health care workers: A meta-analysis of randomized controlled trials
Source: PLoS One. 2025 May 13;20(5):e0321511. doi: 10.1371/journal.pone.0321511 (PMC12074600; doi:10.1371/journal.pone.0321511)
Supplement: S1 File — (DOCX) [file pone.0321511.s003.docx]

**S1 File. Search Strategy**

**PubMed**

1.“COVID-19”[Mesh] OR "COVID-19"[tiab] OR “COVID 19” [tiab] OR “2019-nCoV Infection” [tiab] OR “2019 nCoV Infection” [tiab] OR “2019-nCoV Infections” [tiab] OR “Infection, 2019-nCoV” [tiab] OR “SARS-CoV-2 Infection” [tiab] OR “Infection, SARS-CoV-2” [tiab] OR “SARS CoV 2 Infection” [tiab] OR “SARS-CoV-2 Infections” [tiab] OR “2019 Novel Coronavirus Disease” [tiab] OR “2019 Novel Coronavirus Infection” [tiab] OR “COVID-19 Virus Infection” [tiab] OR “COVID 19 Virus Infection” [tiab] OR “COVID-19 Virus Infections” [tiab] OR “Infection, COVID-19 Virus” [tiab] OR “Virus Infection, COVID-19” [tiab] OR “COVID19” [tiab] OR “Coronavirus Disease 2019” [tiab] OR “Disease 2019, Coronavirus” [tiab] OR “Coronavirus Disease-19” [tiab] OR “Coronavirus Disease 19” [tiab] OR “Severe Acute Respiratory Syndrome Coronavirus 2 Infection” [tiab] OR “COVID-19 Virus Disease” [tiab] OR “COVID 19 Virus Disease” [tiab] OR “Disease, COVID-19 Virus” [tiab] OR “Virus Disease, COVID-19” [tiab] OR “SARS Coronavirus 2 Infection” [tiab] OR “2019-nCoV Disease” [tiab] OR “2019 nCoV Disease” [tiab] OR “2019-nCoV Diseases” [tiab] OR “Disease, 2019-nCoV” [tiab] OR “COVID-19 Pandemic” [tiab] OR “COVID 19 Pandemic” [tiab] OR “Pandemic, COVID-19” [tiab] OR “COVID-19 Pandemics” [tiab]

2."Venous Thromboembolism"[Mesh] OR (Venous Thromboembolism[tiab]) OR (Thromboembolism, Venous[tiab]) OR (VTE[tiab])

3."Venous Thrombosis"[Mesh] OR (Venous Thrombosis[tiab]) OR (Phlebothrombosis[tiab]) OR (Phlebothromboses[tiab]) OR (Thrombosis, Venous[tiab]) OR (Thromboses, Venous[tiab]) OR (Venous Thromboses[tiab]) OR (Deep Vein Thrombosis[tiab]) OR (Deep Vein Thromboses[tiab]) OR (Thromboses, Deep Vein[tiab]) OR (Vein Thromboses, Deep[tiab]) OR (Vein Thrombosis, Deep[tiab]) OR (Deep-Venous Thrombosis[tiab]) OR (Deep-Venous Thromboses[tiab]) OR (Thromboses, Deep-Venous[tiab]) OR (Thrombosis, Deep-Venous[tiab]) OR (Deep-Vein Thrombosis[tiab]) OR (Deep-Vein Thromboses[tiab]) OR (Thromboses, Deep-Vein[tiab]) OR (Thrombosis, Deep-Vein[tiab]) OR (Thrombosis, Deep Vein[tiab]) OR (Deep Venous Thrombosis[tiab]) OR (Deep Venous Thromboses[tiab]) OR (Thromboses, Deep Venous[tiab]) OR (Thrombosis, Deep Venous[tiab]) OR (Venous Thromboses, Deep[tiab]) OR (Venous Thrombosis, Deep[tiab]) OR (DVT[tiab])

4."Pulmonary Embolism"[Mesh] OR (Pulmonary Embolism[tiab]) OR (Pulmonary Embolisms[tiab]) OR (Embolism, Pulmonary[tiab]) OR (Embolisms, Pulmonary[tiab]) OR (Pulmonary Thromboembolisms[tiab]) OR (Pulmonary Thromboembolism[tiab]) OR (Thromboembolism, Pulmonary[tiab]) OR (Thromboembolisms, Pulmonary[tiab]) OR (PE[tiab])

5.#2 OR #3 OR #4

6.#1 AND#5

7.((compar*[tiab]) OR ((singl*[tiab] OR doubl*[tiab] OR tripl*[tiab]) and (mask*[tiab] OR blind*[tiab]))) OR (random*[tiab] OR placebo[tiab] OR controlled[tiab] OR trial*[tiab])

8.#6 AND #7

**Cochrane**

1.MeSH descriptor: [COVID-19] explode all trees

2.(COVID-19 OR COVID 19 OR 2019 nCoV Infection OR SARS-CoV-2 Infection OR Infection, SARS-CoV-2 OR SARS CoV 2 Infection OR SARS-CoV-2 Infections OR 2019 Novel Coronavirus Disease OR 2019 Novel Coronavirus Infection OR COVID-19 Virus Infection OR COVID 19 Virus Infection OR COVID-19 Virus Infections OR Infection, COVID-19 Virus OR Virus Infection, COVID-19 OR COVID19 OR Coronavirus Disease 2019 OR Disease 2019, Coronavirus OR Coronavirus Disease-19 OR Coronavirus Disease 19 OR Severe Acute Respiratory Syndrome Coronavirus 2 Infection OR COVID-19 Virus Disease OR COVID 19 Virus Disease OR COVID-19 Virus Diseases OR Disease, COVID-19 Virus OR Virus Disease, COVID-19 OR SARS Coronavirus 2 Infection OR 2019 nCoV Disease OR COVID-19 Pandemic OR COVID 19 Pandemic OR Pandemic, COVID-19 OR COVID-19 Pandemics):ti,ab

3.#1 OR #2

4.MeSH descriptor: [Venous Thromboembolism] explode all trees

5.("Venous Thromboembolism" OR Venous Thromboembolism OR Thromboembolism, Venous OR VTE):ti,ab

6.#4 OR #5

7.MeSH descriptor: [Venous Thrombosis] explode all trees

8.("Venous Thrombosis" OR Venous Thrombosis OR Phlebothrombosis OR Phlebothromboses OR Thrombosis, Venous OR Thromboses, Venous OR Venous Thromboses OR Deep Vein Thrombosis OR Deep Vein Thromboses OR Thromboses, Deep Vein OR Vein Thromboses, Deep OR Vein Thrombosis, Deep OR Deep-Venous Thrombosis OR Deep-Venous Thromboses OR Thromboses, Deep-Venous OR Thrombosis, Deep-Venous OR Deep-Vein Thrombosis OR Deep-Vein Thromboses OR Thromboses, Deep-Vein OR Thrombosis, Deep-Vein OR Thrombosis, Deep Vein OR Deep Venous Thrombosis OR Deep Venous Thromboses OR Thromboses, Deep Venous OR Thrombosis, Deep Venous OR Venous Thromboses, Deep OR Venous Thrombosis, Deep OR DVT):ti,ab

9.#7 OR #8

10.MeSH descriptor: [Pulmonary Embolism] explode all trees

11.("Pulmonary Embolism" OR Pulmonary Embolism OR Pulmonary Embolisms OR Embolism, Pulmonary OR Embolisms, Pulmonary OR Pulmonary Thromboembolisms OR Pulmonary Thromboembolism OR Thromboembolism, Pulmonary OR Thromboembolisms, Pulmonary OR PE):ti,ab

12.#10 OR #11

13.#6 OR #9 OR #12

14.#3 AND #13

15.((compar*) OR ((singl* or doubl* or tripl*) and (mask* or blind*))) OR (random* or placebo or controlled or trial*):ti,ab

16.#14 AND #15

**Embase**

1.'coronavirus disease 2019'/exp

2.((Covid-19) OR (Covid 19) OR (2019-nCoV Infection) OR (SARS-CoV-2 Infections)):ti,ab

3.#1 OR #2

4.'venous thromboembolism'/exp OR 'venous thromboembolism'

5.((Venous Thromboembolism) OR (Venous Thromboembolism) OR (Thromboembolism, Venous) OR (VTE)):ti,ab

6.#4 OR #5

7.'deep vein thrombosis'/exp OR 'deep vein thrombosis'

8.((Venous Thrombosis) OR (Venous Thrombosis) OR (Phlebothrombosis) OR (Phlebothromboses) OR (Thrombosis, Venous) OR (Thromboses, Venous) OR (Venous Thromboses) OR (Deep Vein Thrombosis) OR (Deep Vein Thromboses) OR (Thromboses, Deep Vein) OR (Vein Thromboses, Deep) OR (Vein Thrombosis, Deep) OR (Deep-Venous Thrombosis) OR (Deep-Venous Thromboses) OR (Thromboses, Deep-Venous) OR (Thrombosis, Deep-Venous) OR (Deep-Vein Thrombosis) OR (Deep-Vein Thromboses) OR (Thromboses, Deep-Vein) OR (Thrombosis, Deep-Vein) OR (Thrombosis, Deep Vein) OR (Deep Venous Thrombosis) OR (Deep Venous Thromboses) OR (Thromboses, Deep Venous) OR (Thrombosis, Deep Venous) OR (Venous Thromboses, Deep) OR (Venous Thrombosis, Deep) OR (DVT)):ti,ab

9.#7 OR #8

10.'lung embolism'/exp OR 'lung embolism'

11.((Pulmonary Embolism) OR (Pulmonary Embolism) OR (Pulmonary Embolisms) OR (Embolism, Pulmonary) OR (Embolisms, Pulmonary) OR (Pulmonary Thromboembolisms) OR (Pulmonary Thromboembolism) OR (Thromboembolism, Pulmonary) OR (Thromboembolisms, Pulmonary) OR (PE)):ti,ab

12.#10 OR #11

13.#6 OR #9 OR #12

14.#3 AND #13

15.compar* OR ((singl* OR doubl* OR tripl*) AND (mask* OR blind*)) OR random*:ti,ab OR placebo:ti,ab OR controlled:ti,ab OR trial*:ti,ab

16.#14 AND #15

**Web of Science**

1.TS=(COVID-19 OR COVID 19 OR 2019-nCoV Infection OR 2019 nCoV Infection OR 2019-nCoV Infections OR Infection, 2019-nCoV OR SARS-CoV-2 Infection OR Infection, SARS-CoV-2 OR SARS CoV 2 Infection OR SARS-CoV-2 Infections OR 2019 Novel Coronavirus Disease OR 2019 Novel Coronavirus Infection OR COVID-19 Virus Infection OR COVID 19 Virus Infection OR COVID-19 Virus Infections OR Infection, COVID-19 Virus OR Virus Infection, COVID-19 OR COVID19 OR Coronavirus Disease 2019 OR Disease 2019, Coronavirus OR Coronavirus Disease-19 OR Coronavirus Disease 19 OR Severe Acute Respiratory Syndrome Coronavirus 2 Infection OR COVID-19 Virus Disease OR COVID 19 Virus Disease OR COVID-19 Virus Diseases OR Disease, COVID-19 Virus OR Virus Disease, COVID-19 OR SARS Coronavirus 2 Infection OR 2019-nCoV Disease OR 2019 nCoV Disease OR 2019-nCoV Diseases OR Disease, 2019-nCoV OR COVID-19 Pandemic OR COVID 19 Pandemic OR Pandemic, COVID-19 OR COVID-19 Pandemics )

2.TS=(Venous Thromboembolism OR Venous Thromboembolism OR Thromboembolism, Venous OR VTE)

3.TS=(Venous Thrombosis OR Venous Thrombosis OR Phlebothrombosis OR Phlebothromboses OR Thrombosis, Venous OR Thromboses, Venous OR Venous Thromboses OR Deep Vein Thrombosis OR Deep Vein Thromboses OR Thromboses, Deep Vein OR Vein Thromboses, Deep OR Vein Thrombosis, Deep OR Deep-Venous Thrombosis OR Deep-Venous Thromboses OR Thromboses, Deep-Venous OR Thrombosis, Deep-Venous OR Deep-Vein Thrombosis OR Deep-Vein Thromboses OR Thromboses, Deep-Vein OR Thrombosis, Deep-Vein OR Thrombosis, Deep Vein OR Deep Venous Thrombosis OR Deep Venous Thromboses OR Thromboses, Deep Venous OR Thrombosis, Deep Venous OR Venous Thromboses, Deep OR Venous Thrombosis, Deep OR DVT)

4.TS=(Pulmonary Embolism OR Pulmonary Embolism OR Pulmonary Embolisms OR Embolism, Pulmonary OR Embolisms, Pulmonary OR Pulmonary Thromboembolisms OR Pulmonary Thromboembolism OR Thromboembolism, Pulmonary OR Thromboembolisms, Pulmonary OR PE)

5.#2 OR #3 OR #4

6.#1 AND #5

7.TS=(((compar*) OR ((singl* or doubl* or tripl*) and (mask* or blind*))) OR (random* or placebo or controlled or trial*))

8.#6 AND #7
